# Self‐criticism and dependency in adolescents with depression: Associations with clinical features and psychological functioning

**DOI:** 10.1111/bjc.70038

**Published:** 2026-02-23

**Authors:** Yushi Bai, Nick Midgley, Patrick Luyten

**Affiliations:** ^1^ Division of Psychology and Mental Health, Faculty of Biology, Medicine and Health University of Manchester Manchester UK; ^2^ Child Attachment and Psychological Therapies Research Unit University College London and Anna Freud London UK; ^3^ Research Department of Clinical, Educational and Health Psychology University College London London UK; ^4^ Faculty of Psychology and Educational Sciences University of Leuven Leuven Belgium

**Keywords:** adolescent depression, dependency, gender, psychological functioning, psychopathology, self‐criticism

## Abstract

**Objectives:**

The personality dimensions of self‐criticism and dependency have been shown to confer vulnerability to depression, and psychopathology more broadly, in adults. However, evidence for the roles of these personality dimensions in young people is weaker, particularly because most studies in this area have been conducted in non‐clinical samples. The current study aimed to examine associations among self‐criticism, dependency and a wide array of indices of interpersonal and intrapersonal functioning in a large sample of clinically depressed adolescents.

**Methods:**

The study sample consisted of 465 adolescents with clinical depression. Associations among self‐criticism, dependency and indices of clinical symptoms and intrapersonal functioning (e.g., depression, anxiety, antisocial behaviour, obsessive‐compulsive features, self‐harm and suicidality) and interpersonal functioning (i.e., friendship, family functioning and parenting as reported by adolescents) were examined using correlation and multiple regression analyses.

**Results:**

Consistent with findings in adults, self‐criticism was more consistently associated with indices of maladaptive functioning than dependency. There was also evidence for gender incongruence effects in that self‐criticism in girls and dependency in boys were associated with increased anxiety, obsessive‐compulsive symptoms and suicidal ideation.

**Conclusions:**

This is the first study in a large sample of clinically depressed adolescents to demonstrate that the personality dimensions of self‐criticism and dependency are associated with clinical features and functioning. The implications of these findings for future research on the role of these personality dimensions in adolescent depression are discussed.

**Practitioner points**

- Self‐criticism and dependency increase vulnerability to depression and broader psychopathology in adults, but their roles in young people remain underexplored
- This study expands on research into clinically depressed adolescents, highlighting that adolescents scoring high on self‐criticism are likely to be more vulnerable to depression, a broader range of psychopathologies and impaired social functioning.
- The impacts of self‐criticism and dependency on psychopathology may differ between adolescent girls and boys, underscoring the need for further investigations into the underlying mechanisms
- These findings are crucial for clinical practice and policy, especially given the alarming increase in emotional symptoms in adolescents. Although further research is needed, self‐criticism is a key target for prevention and intervention to improve young people's well‐being.

## INTRODUCTION

Adolescence is a critical developmental period marked by heightened vulnerability to mental health difficulties (Shorey et al., 2022). Estimates suggest that up to 20% of adolescents experience a major depressive episode by the end of this stage (Thapar et al., 2012). Moreover, recent research suggests an escalating mental health crisis among young people across Western and industrialized countries (McGorry et al., 2024). In the United Kingdom, the prevalence of common mental disorders (e.g., depression and anxiety) among young people recorded in primary care has nearly tripled over the past decade (Dykxhoorn et al., 2023). These young people are experiencing lifespan adverse outcomes and consuming extensive health services (Meltzer et al., 2011). It is therefore crucial to identify individual‐level vulnerabilities that may elevate the risk of depression and broader psychopathology in adolescence.

A substantial body of research indicates that self‐criticism and dependency, as two maladaptive personality traits, are associated with an increased risk for depression and psychopathology more broadly in adolescents (e.g., Blatt & Luyten, 2009; Campos et al., 2014; Shahar et al., 2020). In addition, there is a sizeable body of literature showing that self‐criticism is also associated with externalizing symptoms (Campos et al., 2014; Leadbeater et al., 1999). Both dependency and self‐criticism have also been shown to influence features of the social environment, with self‐criticism generally exerting a more detrimental impact on relationships than dependency (e.g., Priel & Shahar, 2000; Shahar, 2015; Shahar & Priel, 2003). Yet, there remains a dearth of studies that have tested the validity of this personality approach in clinical samples. While findings from non‐clinical samples may illuminate potential risk factors prior to clinical onset, stronger evidence derived directly from clinical populations is essential to extend and substantiate these insights. The present study therefore sought to replicate and extend previous findings by examining the role of self‐criticism and dependency in explaining vulnerability not only to internalizing symptoms, including depression, but also to a broader range of associated clinical features in a large clinical sample of young people diagnosed with depression. In addition, we also included a focus on the impact of these personality dimensions on interpersonal functioning, particularly the quality of friendships.

### The role of dependency and self‐criticism in depression and broad psychopathology

Self‐criticism is thought to reflect an overemphasis on self‐definitional issues (Blatt & Zuroff, 1992). Self‐critical individuals tend to exhibit excessive preoccupation with self‐worth and autonomy, which includes deep‐seated feelings of failure, worthlessness and guilt. Dependency, by contrast, involves an overemphasis on interpersonal relatedness, and is characterized by maladaptive concerns regarding interpersonal separation and abandonment. These two personality dimensions are generally assumed to derive from negative early experiences (Blatt, 2004a); studies have suggested that self‐criticism is associated with critical, achievement‐oriented controlling parenting (Bleys et al., 2018), whereas dependency is related to more dependent‐oriented and overprotective controlling parenting (Campos et al., 2010).

The majority of studies regarding the role of these two personality dimensions have focused on mood disorders. Longitudinal studies have shown that both self‐criticism and dependency are associated with elevated levels of depression and anxiety in adult populations and community‐based adolescents (e.g., Kopala‐Sibley et al., 2015; Sherry et al., 2014; Smith et al., 2016; Werner et al., 2019). Self‐criticism has shown a stronger association with depressive symptoms than dependency, whereas findings in relation to anxiety have been more mixed. It has been argued that individuals high on self‐criticism tend to be socially isolated and exhibit traits of expressive suppression and self‐concealment, possibly to protect their vulnerable sense of self and maintain autonomy (Luoma & Chwyl, 2022). These traits may lead to reduced social support and heightened emotional distress (Shahar, 2015). Indeed, Dunkley et al. (2009) followed an adult clinical sample for 4 years and found that the association between self‐criticism in year 1 and depression and psychosocial impairments in year 4 could be explained by the development of a negative perception of social support and social interactions by year 3.

The more consistent negative role of self‐criticism is also reflected in research concerning associations between self‐criticism, dependency and other clinical features. Although studies have shown that self‐criticism and dependency are both implicated in self‐harm (Cohen et al., 2015; Xavier et al., 2017) and suicidality (Fazaa & Page, 2003, 2009; Smith et al., 2018), again, self‐criticism emerged as a stronger factor in these studies. For instance, in a study of 64 college students who had attempted suicide, Fazaa and Page (2003) found that self‐criticism was associated with more lethal suicide attempts. Self‐criticism has also been shown to relate to more externalizing problems, such as aggressive, antisocial and oppositional behaviours (Campos et al., 2014; Vandenkerckhove et al., 2019).

However, as noted, most studies in this area have focused on non‐clinical adolescent samples and have primarily investigated mood disorders. Therefore, further research using clinical samples is needed concerning the purported role of dependency and self‐criticism in explaining vulnerability for depression and a broader range of associated features, including both intrapersonal symptoms and functioning (e.g., internalizing and externalizing symptoms) and interpersonal functioning (e.g., social relationships). Furthermore, much research in this area has neglected the potential role of gender. It has been argued that traditional social norms tend to value attachment and relatedness in women, whereas autonomy and achievement are valued more in men (Blatt & Luyten, 2009). Although campaigns promoting gender equality have achieved substantial progress, systematic reviews show that gender differences in personality and emotion expression persist, even within societies characterized by higher living standards (Herlitz et al., 2024). Women continue to shoulder a disproportionate share of caregiving and emotional labour, a disparity that was further intensified during the COVID‐19 pandemic (Gencer et al., 2024; Sarrasanti et al., 2020; Xue & McMunn, 2021). At the same time, women are increasingly expected to combine these responsibilities with occupational achievement, which has been linked to heightened stress and emotional vulnerability (Ervin et al., 2022; Nilsen et al., 2017). In personality research, it has been proposed that gender incongruence (e.g., higher levels of dependency in men and self‐definition in women) may be associated with increased risk for psychopathology, as gender‐incongruent personality features may be associated with explicit and implicit criticism from others (Blatt, 2004b; Luyten et al., 2007). However, empirical evidence directly examining this assumption remains limited. One recent study reported that women experienced greater depressive symptoms on days when they felt less feminine or more masculine (Yan et al., 2024), yet research specifically examining the gender role on the relation between personality and mental health outcomes still warrants further investigation.

### The present study

Given the limitations in existing research summarized above, the present study aimed to investigate whether self‐criticism and dependency were associated with indices of clinical symptoms and intrapersonal functioning (i.e., depression, anxiety, antisocial behaviour, obsessive‐compulsive features, risk‐taking and self‐harming behaviour, suicidality, global functioning, self‐esteem, rumination and general emotion states) and interpersonal functioning (i.e., parenting, family functioning and friendship as perceived by adolescents) in theoretically expected ways among a large sample of clinically depressed adolescents (*N* = 465). We expected that (a) associations between self‐criticism and indices of maladaptive intrapersonal and interpersonal functioning would be stronger compared with associations with dependency and (b) self‐criticism in girls and dependency in boys would be associated with higher levels of maladaptive functioning, consistent with the gender incongruency hypothesis.

## METHODS

### Participants

The current study drew on data from the Improving Mood with Psychoanalytic and Cognitive Therapies (IMPACT) study (Goodyer et al., 2017), a randomized controlled trial investigating the effectiveness of psychotherapies in adolescents with major depressive disorder. Adolescents aged between 11 and 17 years who met DSM‐IV criteria for major depressive disorder were recruited from 15 National Health Service Child and Adolescent Mental Health Service (CAMHS) clinics across three regions of the United Kingdom: East Anglia, North London and North‐West England (Manchester and the Wirral). Participants were excluded from the study if they (1) had generalized learning difficulties, (2) had a pervasive developmental disorder, (3) were pregnant, (4) were unable to discontinue a medication that could interact with a selective serotonin reuptake inhibitor, (5) were abusing substances or (6) had a primary diagnosis of bipolar type I disorder, schizophrenia or an eating disorder. In total, 470 adolescents were recruited. Five participants withdrew from the study after randomization, leaving 465 adolescents included in the analysis. More details of the inclusion and exclusion criteria, as well as the recruitment and research procedures, have been published elsewhere (Goodyer et al., 2017). All participants provided written informed consent, as did their parents/guardians. Ethical approval was granted by the Cambridgeshire 2 Research Ethics Committee, Addenbrooke's Hospital, Cambridge, UK (reference 09/H0308/137).

To address the current research questions, baseline data from the IMPACT study were used. Participants were aged from 11.30 to 17.99 years (*M* = 15.61, *SD* = 1.42). There were 348 female participants (mean age = 15.72, *SD* = 1.31) and 117 male participants (mean age = 15.28, *SD* = 1.67). The majority of the participants (80.7%) were White and British. Participants' comorbid diagnoses were screened by a semi‐structured interview measurement of Kiddie‐Schedule for Affective Disorders and Schizophrenia Present and Lifetime version (Kaufman et al., 1997). There were 225 participants (48%) who received comorbid psychiatric diagnoses. Of these, 86.67% of participants had less than two comorbidities. The most frequent comorbidities that account for over 80% of diagnoses were generalized anxiety disorder, social phobia, oppositional defiant disorder, specific phobia, post‐traumatic stress disorder and separation anxiety disorder.

### Measures

#### Personality dimensions

##### Self‐criticism and dependency

The short version of the Depressive Experiences Questionnaire for Adolescents (DEQ‐A; Fichman et al., 1994) was used to assess self‐criticism and dependency in the current study. The DEQ‐A short version is a 20‐item questionnaire assessing self‐criticism (8 items), dependency (8 items) and efficacy (4 items) using a 7‐point Likert‐type scale. For this study, only results for the self‐criticism and dependency subscales are reported. To the best of our knowledge, no study has investigated the factor structure of the DEQ‐A short version in a clinical sample. We therefore evaluated its factor structure using confirmatory factor analysis (CFA). Full details of the analytical procedure and results are provided in Appendix S1. Maximum likelihood estimation was adopted, and the evaluation and comparison of model fits were based on the multiple criteria of the goodness‐of‐fit index (i.e., absolute fit and incremental fit indices). After deleting item 2 from the Dependency subscale and item 19 from the Self‐criticism subscale, a two‐factor solution showed an acceptable and better model fit compared with a one‐factor model and also demonstrated measurement invariance across sex. Furthermore, both modified subscales exhibited adequate internal consistency, with Cronbach's alpha of .669 and .721 for self‐criticism and dependency, respectively.

#### Indices of intrapersonal functioning

##### Depression

The Mood and Feelings Questionnaire (MFQ; Costello & Angold, 1988) was used to assess depression. This 3‐point, 33‐item questionnaire was designed according to the DSM‐IV criteria and measures depressive symptoms among youths aged between 6 and 16 years.

##### Anxiety

The Revised Children's Manifest Anxiety Scale (RCMAS; Reynolds & Richmond, 1978) is a 3‐point, 28‐item scale measuring the general anxiety of children and adolescents, including physiological anxiety, worry or over sensitivity, and social concerns.

##### Obsessive‐compulsive symptoms

The Short Leyton Obsessional Inventory (LOI; Bamber et al., 2002), a 3‐point, 11‐item scale, was used to measure obsessive‐compulsive symptoms (e.g., obsessional thinking and compulsive behaviours) in the study sample.

##### Antisocial behaviour

The Behaviour Checklist (BC; Goodyer et al., 2017), a 3‐point, 11‐item checklist, was used to gather data on participants' current antisocial behaviour. It is designed based on the DSM‐IV criteria for conduct and oppositional disorders.

##### Risk‐taking and self‐harming behaviour

The Risk‐Taking and Self‐Harming Inventory for Adolescents (RTSHIA; Vrouva et al., 2010), a 4‐point, 26‐item scale that yields two subscales of Risk‐taking (RT) and Self‐harm (SH), was applied to assess both risk‐taking behaviours (e.g., smoking and gang violence) and self‐harm behaviours (e.g., picking at wounds, overdose).

##### Suicidal ideation and behaviour

The Columbia Suicide Severity Rating Scale (C‐SSRS; Posner et al., 2011) was used to assess suicidal ideation and suicidal behaviours. The current study regarded the suicidal ideation (SI) subscale as a 5‐point ordinal scale comprising the following categories: (1) wish to die; (2) non‐specific active suicidal thoughts; (3) active suicidal ideation with methods without intent to act; (4) active suicidal ideation with some intent to act, without a specific plan; and (5) active suicidal ideation with a specific plan and intent. Similarly, the suicidal behaviour (SB) subscale is regarded as a 4‐point ordinal scale made up of the following categories: (1) preparatory acts or behaviour; (2) aborted attempt; (3) interrupted attempt; and (4) actual attempt (non‐fatal). The absence of suicidal ideation or behaviour was coded as 0. The C‐SSRS captures both present and lifetime suicidal ideation and behaviour. In the current study, ‘present’ referred to the current episode of major depression, while ‘lifetime’ referred to the entire span of the participant's life, excluding the current episode.

##### General health and social functioning

The Health of the Nation Outcome Scales for Children and Adolescents (HoNOSCA; Gowers et al., 1999) is a 4‐point, 13‐items scale assessing general mental health and social functioning, including symptomatic, behavioural, social and impairment domains.

##### Rumination

The Ruminative Responses Scale (RRS; Nolen‐Hoeksema & Morrow, 1991), a 4‐point, 21‐item scale, was used to assess the ruminative response style to low mood. Specifically, this scale measures to what extent the response style is focused on self, symptoms and possible consequences and causes of low moods.

##### Self‐esteem

The Rosenberg Self‐esteem Scale (RSES; Rosenberg, 1965), a widely used 4‐point, 10‐item measure, was used to measure global self‐esteem.

##### Positive and negative emotions

The Differential Emotions Scale‐IV (DES‐IV; Boyle, 1984) was used to assess fundamental mood states. This 36‐item, 5‐point scale consists of two indexes: Positive Emotions (i.e., interest, joy and surprise) and Negative Emotions (i.e., anger, disgust, contempt, self‐hostility, fear, shame, shyness and guilt) (Youngstrom & Green, 2003).

#### Indices of interpersonal functioning

##### Parenting

The Alabama Parenting Questionnaire‐9 (APQ‐9; Elgar et al., 2007) was used to assess how adolescent participants perceived their parents' parenting practices. This 5‐point, 9‐item scale consists of three subscales, namely Positive Parenting (PP), Inconsistent Discipline (ID) and Poor Supervision (PS).

##### Family functioning

The Family Assessment Device – General Functioning (FAD‐GF; Epstein et al., 1983), a 4‐point, 12‐item scale, was used to report overall family functioning as perceived by the participants.

##### Friendships

The Friendship Questionnaire (FQ; Goodyer et al., 2017), a 4‐point, 8‐item scale, was used to assess the number and quality of participants' friendships.

All of the measures were self‐reports, with higher scores reflecting higher levels of measured functioning/symptoms. The internal consistency of the measures was tested, and a Cronbach's alpha larger than .65 was considered to be adequate (Vaske, 2008). In the current study, values of Cronbach's alpha ranged from .59 to .92 (*M* = .79, *SD* = .10). Only the HoNOSCA had a Cronbach's alpha (.59) lower than .65. The fairly poor internal consistency of the HoNOSCA has been reported previously (Harnett et al., 2005) and can be interpreted as resulting from the fact that this scale captures functioning across a number of independent psychological domains.

### Statistical analysis

Pearson correlations and multiple regression were used to investigate associations between self‐criticism and dependency, and their interactions with each other and with sex, and indices of intrapersonal and interpersonal functioning. For continuous variables, Pearson's correlation and multiple linear regression were used. For ordinal scales (i.e., C‐SSRS), Spearman's correlation and ordinal regression were used. Given that 89.9% (*N* = 418) of the participants reported the absence of present suicidal behaviours, binary logistic regression was used for the C‐SSRS subscale of present suicidal behaviour. The standardized beta (*β*) and odds ratio (OR) were reported in regression analyses to reflect the impact of predictor variables on dependent variables. All the analyses were conducted using SPSS version 25.

### Missing data

A total of 16.99% of data was missing for the DEQ‐A, with an average of 20.92% missing data across the other measures. To assess the potential impact of missing data, we compared participants who provided complete data and those with missing data on key study‐related variables (dependency, self‐criticism and depressive symptoms), following the suggestions of Enders (2010). Across all comparisons, results were non‐significant and effect sizes were small (Cohen's *d* < .20), suggesting minimal systematic differences between the two groups. Given the lack of significant differences and the limited extent of missingness, we proceeded with complete‐case analysis.

## RESULTS

### Correlational analyses

Table 1 shows zero‐order correlations among the study variables. As expected, self‐criticism was more consistently and more strongly related to clinical symptoms (e.g., depression, suicidal ideation and behaviour, impairment in general functioning) and maladaptive functioning (e.g., impaired self‐esteem) compared with dependency overall. Interestingly, when examining correlations by sex, dependency was associated with more maladaptive functioning among boys, whereas no marked differences were observed for self‐criticism, which may reflect gender incongruence patterns.

**TABLE 1:** Zero‐order correlations for self‐criticism, dependency and functioning.

	Self‐criticism			Dependency		
	Whole sample	Girls	Boys	Whole sample	Girls	Boys
Personality vulnerability						
Self‐criticism	–	–	–	.**51** **	.**50** **	.**50** **
Interpersonal functioning						
Family functioning (FAD)	.**33** **	.**30** **	.**46** **	.**16** *	.11	.**39** *
Parenting – Positive parenting (APQ)	**−.20** **	**−.18** *	**−.31** *	−.03	.02	**−.25** *
Parenting – Inconsistent discipline (APQ)	.12	.12	.1	.11	.13	.03
Parenting – poor supervision (APQ)	.**21** **	.**20** **	.**31** *	.**15** *	.11	.**36** **
Friendship (FQ)	−.08	−.06	−.12	.05	.1	−.09
Intrapersonal functioning – clinical expressions						
Depression (MFQ)	.**49** **	.**48** **	.**50** **	.**29** **	.**24** **	.**35** **
Anxiety (RCMAS)	.**32** **	.**33** **	.**21** *	.**34** **	.**28** **	.**41** **
Obsessional behaviour (LOI)	.**25** **	.**29** **	.1	.**28** **	.**28** **	.**26** **
Antisocial behaviour (BC)	.08	.**12** *	.11	**−.11** *	−.03	−.18
Risk‐taking behaviour (RTSHIA)	.06	.07	.04	.**15** **	.**19** **	.07
Self‐harming behaviour (RTSHIA)	0	.04	−.07	.04	.1	−.09
General health and social functioning (HoNOSCA)	.**16** **	.**14** *	.**23** *	−.03	0	−.12
Present suicidal ideation (C‐SSRS)^a^	.**16** *	.09	.**29** *	.06	−.05	.**30** *
Lifetime suicidal ideation (C‐SSRS)^a^	.**14** *	.11	.16	.**19** **	.**18** *	.14
Present suicidal behaviour (C‐SSRS)^a^	.**13** *	.11	.15	.06	.06	0
Lifetime suicidal behaviour (C‐SSRS)^a^	.**26** **	.**25** **	.19	.**25** **	.**24** **	.18
Intrapersonal functioning – personal attributes						
Rumination (RRS)	.**46** **	.**42** **	.**51** **	.**43** **	.**37** **	.**51** **
Self‐esteem (RSES)	**−.58** **	**−.55** **	**−.60** **	**−.33** **	**−.26** **	**−.38** **
Positive emotions (DES)	.06	−.02	.22	−.02	−.05	.06
Negative emotions (DES)	.06	.05	.11	.02	−.04	.19

*Note*: Bold indicates for statistical significant values.

^a^ Only C‐SSRS used Spearman's correlation whereas others used Pearson's correlation.

* *p* < .05.

** *p* < .001.

### Regression analyses for clinical symptoms and intrapersonal functioning

Table 2 presents the regression model results for clinical symptoms and intrapersonal functioning. As expected, self‐criticism was associated with depressive symptoms (*β* = .420, *p* < .001), antisocial behaviour symptoms (*β* = .324, *p* = .007), general and social functioning (*β* = .395, *p* = .002), and self‐esteem issues (*β* = −.562, *p* < .001). Dependency was not associated with depressive symptoms (*β* = .118, *p* = .272) or self‐esteem (*β* = −.103, *p* = .302), but was significantly associated with reduced antisocial behaviours (*β* = −.370, *p* = .002) and less impairment in general and social functioning (*β* = −.308, *p* = .013). Both self‐criticism and dependency were significantly associated with lifetime suicidal behaviour (OR = 1.36 and 1.20, respectively) and rumination (*β* = .340 and .347, respectively). None of them was associated with risk‐taking and self‐harming behaviour, emotional experiences, lifetime suicidal ideation or current suicidal behaviour.

**TABLE 2:** Regression analysis for variables predicting clinical expressions.

	MFQ			RCMAS			LOI			BC			RTSHIA‐RT			RTSHIA‐SH			HoNOSCA		
	*B*	*SE B*	*β*	*B*	*SE B*	*β*	*B*	*SE B*	*β*	*B*	*SE B*	*β*	*B*	*SE B*	*β*	*B*	*SE B*	*β*	*B*	*SE B*	*β*
SC	4.97	1.26	.**42** **	−.27	.94	−.03	−.42	.67	−.07	1.11	.41	.**32** **	.23	.69	.04	−.53	1.52	−.04	2.57	.82	.**40** **
D	1.14	1.04	.12	2.60	.77	.**38** **	1.14	.56	.**24** *	−1.04	.34	**−.37** **	.53	.57	.12	−.77	1.26	−.08	−1.64	.66	**−.31** *
Gender	1.67	1.23	.07	1.28	.91	.07	−.03	.66	−.00	−1.19	.40	−.17**	.03	.66	.00	−.57	1.45	−.02	.14	.78	.01
SC × D	−.78	.84	−.08	−.71	.62	−.10	−.45	.45	−.09	.04	.27	.01	.62	.45	.13	−.23	1.00	−.02	.15	.53	.03
SC × gender	.50	1.45	.04	2.36	1.07	.**24** *	1.56	.77	.**23** *	−.56	.47	−.14	−.46	.79	−.07	.36	1.74	.03	−1.34	.93	−.18
D × gender	−1.10	1.19	−.10	−1.55	.89	−.19	−.24	.64	−.04	.76	.39	.23	.43	.65	.08	1.78	1.44	.15	1.14	.76	.18
SC × D × gender	.05	1.02	.00	.26	.76	.03	.27	.54	.05	.21	.33	.06	−.97	.55	−.16	−.71	1.22	−.05	.26	.64	.04
*F*/*χ* ^2^	**19.98** **			**10.80** **			**6.40** **			**4.56** **			1.97			.86			**2.88** **		

	RRS			RSES			DES‐PE			DES‐NE			C‐SSRS SI‐Present	C‐SSRS SI‐Lifetime	C‐SSRS SB‐Present^a^
	B	SE B	β	B	SE B	β	B	SE B	β	B	SE B	β	OR (95% CI)	OR (95% CI)	OR (95% CI)
SC	4.82	1.53	.**34** **	−2.95	.52	**−.56** **	1.37	.88	.27	−.23	3.56	−.01	**1.32** * (1.04, 1.66)	1.03 (.80, 1.34)	1.92 (.50, 7.40)
D	4.04	1.29	.**35** **	−.45	.43	−.1	−.89	.81	−.21	3.91	3.25	.23	.85 (.71, 1.03)	1.19 (.97, 1.47)	.79 (.31, 2.02)
Gender	2.43	1.49	.08	−1.39	.51	−.13**	.19	.89	.02	−.68	3.59	−.02	.9 (.61, 1.32)	.74 (.50, 1.10)	.74 (.25, 2.20)
D × SC	.37	1.02	.03	0	.35	0	−.7	.55	−.16	1.27	2.22	.07	1.12 (.92, 1.36)	1.08 (.86, 1.36)	.59 (.17, 2.13)
D × gender	−1.62	1.47	−.12	.48	.5	.09	.63	.91	.13	−5.24	3.66	−.27	1 (.64, 1.57)	1.19 (.75, 1.88)	.82 (.19, 3.56)
SC × gender	−.35	1.76	−.02	.15	.6	.02	−1.36	1.04	−.22	2.29	4.18	.09	**1.62** * (1.10, 2.39)	.89 (.60, 1.30)	1.27 (.44, 3.64)
D × SC × gender	−.69	1.24	−.05	.48	.42	.09	.27	.69	.05	−.13	2.78	−.01	.96 (.68, 1.35)	.88 (.63, 1.23)	1.76 (.45, 6.80)
*F*/*χ* ^2^	**19.20** **			**29.58** **			.9			.5			**17.51** *	13.82	**7.87**

*Note*: The reference category was ‘girl’ for ‘gender’. Bold indicates for statistical significant values.

Abbreviations: *B*/*β* = unstandardized/standardized regression coefficient; BC, Behaviours Checklist; CI, confidence interval; C‐SSRS, Classification Suicide Severity Rating Scale; DES‐PE/NE, Differential Emotion Scale – Positive Emotion/Negative Emotion; HoNOSCA, Health of the Nation Outcome Scale for Children and Adolescents; LOI, Leyton Obsessional Inventory; MFQ, Mood and Feelings Questionnaire; OR, odds ratio; RCMAS, Revised Children's Manifest Anxiety Scale; RRS, Ruminative Responses Scale; RSES, Rosenberg's Self‐esteem Scale; RTSHIA‐RT/SH, The Risk‐Taking and Self‐Harming Inventory for Adolescents – Risk‐Taking/Self‐Harm; S/D, self‐criticism/dependency from modified DEQ‐A; SB, suicidal behaviour; SE, standard error; SI, suicidal ideation.

^a^ For C‐SSRS sub‐scles, models analysed by ordinal regression, except the model predicting SB‐Present, which adopted binary logistic regression. Its model fit was based on Hosmer and Lemeshow test with a non‐significant result indicating a good model fit.

* *p* < .05.

** *p* < .001.

The interaction between self‐criticism and dependency was non‐significant in all regression analyses. However, significant interactions with sex were detected for self‐criticism in the prediction of anxiety (*β* = .243, *p* = .028) and obsessive‐compulsive symptoms (*β* = .232, *p* = .044). Dependency, on the other hand, significantly interacted with sex on present suicidal ideation (OR = 1.62, *p* = .014).

To better interpret the interactions among self‐criticism, dependency and sex, further regression analyses of the three clinical symptoms (i.e., anxiety, obsessive‐compulsive symptoms and present suicidal ideation) were conducted separately and in parallel for girls and boys (Table 3). Gender incongruence patterns were then observed. Self‐criticism was significantly associated with anxiety (*β* = .254, *p* < .001), obsessive‐compulsive symptoms (*β* = .194, *p* = .003) and present suicidal ideation (OR = 1.32, *p* = .018) only among girls. By contrast, dependency showed a stronger significant effect on anxiety (RCMAS, *β* = .373, *p* = .001) and obsessive‐compulsive symptoms (LOI, *β* = .252, *p* = .038) among boys compared with girls (for RCMAS, *β* = .153, *p* = .016; for LOI, *β* = .185, *p* = .004). In addition, dependency tended to be associated with an increased risk of present suicidal ideation among boys (OR = 1.37, *p* = .069) compared with girls (OR = .85, *p* = .090).

**TABLE 3:** Regression analysis for variables predicting RCMAS, LOI and present C‐SSRS‐SI by gender.

	RCMAS						LOI						C–SSRS–SI–present^a^	
	Girls			Boys			Girls			Boys			Girls	Boys
	*B*	*SE B*	*β*	*B*	*SE B*	*β*	*B*	*SE B*	*β*	*B*	*SE B*	*β*	OR (95% CI)	OR (95% CI)
SC	2.09	.52	.**25** **	−.27	.95	−.03	1.15	.38	.**19** **	−.42	.66	−.08	**1.32** * (1.05, 1.66)	1.30 (.88, 1.93)
D	1.05	.43	.**15** *	2.60	.79	.**37** **	.91	.32	.**19** **	1.14	.54	.**25** *	.85 (.71, 1.03)	1.37 (.98, 1.93)
SC × D	−.45	.43	−.06	−.71	.64	−.12	−.17	.31	−.03	−.45	.44	−.12	1.12 (.93, 1036)	1.07 (.80, 1.42)
*F*/*χ* ^2^	**14.59** **			**6.58** **			**11.59** **			2.68			**8.11** *	6.63

*Note*: Bold indicates for statistical significant values.

Abbreviations: *B*/*β*, unstandardized/standardized regression coefficient; CI, confidence interval; C‐SSRS‐SI, Classification Suicide Severity Rating Scale – suicidal ideation; LOI, Leyton Obsessional Inventory; OR, odds ratio; RCMAS, Revised Children's Manifest Anxiety Scale; S/D, self‐criticism/dependency from modified DEQ‐A; SE, standard error.

^a^ Only C‐SSRS‐SI‐Present used ordinal regression analysis, while others used linear regression analysis.

* *p* < .05.

** *p* < .001.

### Regression analyses for interpersonal functioning

Finally, we investigated the associations between personality variables and interpersonal functioning (i.e., parenting, family functioning and friendship as perceived by adolescents). Self‐criticism was associated only with adolescents' perceived family functioning (*β* = .29, *p* = .005), suggesting that the greater the extent to which family functioning was perceived by the young person as being maladaptive, the higher their levels of self‐criticism were. None of the other regression models was significant. Detailed results are provided in Tables S2 and S3.

## DISCUSSION

To our knowledge, this is the first study to investigate the roles that self‐criticism and dependency, including their interaction with sex, play in a wide range of indices of interpersonal and intrapersonal functioning by using a large sample of clinically depressed adolescents. Four major sets of findings stand out.

First, the findings indicate a more prominent and detrimental impact of self‐criticism on clinical symptoms and functioning compared with dependency. Self‐criticism was determined to be a more robust risk factor for depression, as dependency was no longer significantly associated with depressive symptoms after self‐criticism was introduced into the regression model. This finding is consistent with previous evidence that suggests self‐criticism is more consistently associated with more severe depressive symptoms in adults (Luyten et al., 2007; Smith et al., 2016). One possibility is that individuals with self‐criticism may actively generate a more negative environment by assimilating life events into a self‐critical schema (Blatt, 2004a). For example, Priel and Shahar (2000) followed 182 adults for 9 weeks. Whereas baseline dependency predicted later distress only after interpersonal stress, self‐criticism was associated with increased distress over time. Moreover, it has previously been discussed that self‐criticism tends to generate a negative social context with lower levels of perceived social support (Dunkley et al., 2009). On the other hand, dependency is characterized by intense fears of being abandoned and loneliness; therefore, individuals with dependent traits may tend to put considerable effort into establishing and maintaining close relationships and avoiding confrontations (Santor & Zuroff, 1998). Such features may benefit those individuals so that they receive more social support than self‐critical individuals. Shahar and Priel (2003), for instance, followed 603 adolescents over 17 weeks to investigate their generated and perceived social environment. Although both dependency and self‐criticism predicted significant increases in negative life events, which, in turn, increased depressive symptoms, dependency also predicted significant increases in positive life events. Therefore, self‐criticism seems to be a consistent vulnerability factor, whereas dependency seems to encompass both risk factors and protective factors (i.e., social support). This assumption may also help to explain previous findings on dependency, which propose that it may be deleterious only when individuals have at‐risk social support systems (Adams et al., 2009).

Consistent with the assumption regarding the different interpersonal patterns of the two expressions, self‐criticism was significantly associated with increased antisocial behaviour, and more impaired general health and social functioning, whereas dependency showed significant preventive effects in relation to both of these. Although these findings might again reflect the prosocial features of dependency, existing research on anger, a concept that is closely correlated with antisocial features (Hawes et al., 2015), may also help to explain the findings. Abi‐Habib and Luyten (2013) investigated 253 adults and found that dependency was associated with a higher tendency to direct anger towards oneself and a lower tendency to direct anger towards others. Conversely, self‐criticism was associated with higher levels of turning anger towards both self and others, with lower anger control. It is possible that increased anger towards others and poor emotion regulation limit self‐critical individuals' capacity to cope with frustrations and amplify their engagement in aggressive and antisocial behaviours.

In line with the theoretical framework, self‐criticism was also determined to be significantly associated with impaired self‐esteem and maladaptive family functioning as perceived by the young people. Both dependency and self‐criticism displayed similar associations with factors that have been implicated in adolescent depression, namely lifetime suicidal behaviour and rumination, consistent with previous research (Fehon et al., 2000; Spasojević & Alloy, 2001). It is possible that the overly high standards in self‐criticism or the fears of loneliness and abandonment in dependency activate self‐evaluation concerns, and thus increase the likelihood of engaging in rumination.

The second set of findings provided evidence to support the gender incongruence effect. Self‐criticism emerged as a stronger risk factor for anxiety, obsessive‐compulsive symptoms and present suicidal ideation among girls than in boys, while the opposite pattern was observed for dependency. It has been proposed that having gender‐incongruent personality features may lead to implicit and explicit external criticisms, which, in turn, increase the risk of psychopathology (Luyten et al., 2007). If this assumption holds, the effect of gender incongruence would be expected to be more pronounced in disorders closely related to external criticism. Indeed, Pace et al. (2011) argued that two key domains of obsessive‐compulsive disorder—inflated responsibility and socially prescribed perfectionism—are driven by the desire for approval from others and the avoidance of external criticism. Bhar and Kyrios (1999) analysed 152 non‐clinical adults and found that socially prescribed perfectionism, a concept reflecting concerns about evaluations by others, was significantly associated with obsessive‐compulsive symptoms after controlling for depressive symptoms. It is possible that gender‐incongruent features may be deleterious only when they lead to external judgement and/or criticism, which may help to explain the inconsistent reports on the gender incongruence effect in previous research.

Third, self‐criticism and dependency exhibited weak associations with self‐harming, risk‐taking behaviour and parenting styles perceived by participants. The short‐form instruments that were used in this study may have influenced these findings. For example, although the nine‐item, three‐subscale APQ‐9 (Elgar et al., 2007) could provide an overall profile of parenting styles (e.g., positive vs. negative parenting), it may have limited capacity to capture specific types of problematic parenting (e.g., achievement‐oriented vs. dependent‐oriented parenting) and this may weaken its effects on regression models for self‐criticism and dependency.

Finally, friendship and emotional states were not associated with self‐criticism or dependency in the current analysis. This finding may reflect differences between clinical and non‐clinical samples, as studies that reported significant associations with friendship and interpersonal relationships primarily used samples of college students. Another possibility is that the questionnaires that were used reflect only the general patterns of participants' functioning. For example, item 2 in the 8‐item FQ (Goodyer et al., 2017) asked participants how often they see their friends. Although such an item is telling in relation to participants' social resources, the FQ's exclusive focus on friendships may weaken its capacity to assess the quality of social support or interactions in depth, which may lead to the gap between the current findings and previous findings regarding a more comprehensive concept of social support.

The present findings yield several clinical implications. First, by using a large sample of adolescents diagnosed with depression, the present study highlights the association between self‐criticism and depression as well as a broader range of psychopathologies, including anxiety, antisocial behaviour, obsessive‐compulsive symptoms and suicidality. Young people with self‐criticism are likely to be more vulnerable to developing clinical symptoms and experiencing impaired social functioning. These results underscore the importance of targeting self‐criticism as a key risk factor in adolescents with depression. To develop effective prevention and intervention strategies for this group, further research is needed to clarify the mechanisms underlying this association. Second, this study provides evidence to support the gender incongruence effect. Although further investigations into the underlying mechanisms are needed, clinicians and researchers are recommended to consider the potential differences in the impacts of the two personality dimensions between boys and girls, particularly when working with young people in environments with more traditional views on gender roles.

When interpreting the findings, several limitations need to be considered. The study adopted a cross‐sectional design; therefore, it was not possible to determine causal relationships among variables. Moreover, although a wide range of clinical conditions and functioning were included, all of the analyses were based on a sample diagnosed with moderate to severe depression. Thus, the potential impacts of the nature of the sample should be considered. Furthermore, given that a wide range of psychological functioning was investigated, the IMPACT study used several brief measures to ensure time efficiency. As noted earlier, brief measures may not be sufficiently sensitive to detect subtle but meaningful variation in expressions of functioning, which might limit our capacity to further elaborate on the results. Nevertheless, we believe that the findings are promising in providing fairly comprehensive profiles of self‐criticism and dependency among adolescents that take sex into consideration. Such findings demonstrate the value of considering adolescent personality vulnerabilities in research on psychopathology. Further studies are required to investigate the proposed assumptions and further clarify the underlying mechanisms of psychopathology in light of personality vulnerabilities.

## CONCLUSION

To our knowledge, the current study is the first to use a large sample of adolescents diagnosed with depression to evaluate the role of the personality vulnerabilities of dependency and self‐criticism on a wide range of functioning. The results broadly supported our hypotheses and extend previous findings into the clinical adolescent population. The study provides evidence for the role of personality vulnerabilities, especially self‐criticism, on psychopathology and psychosocial functioning. It is noteworthy that the current study observed different patterns in terms of dependency and self‐criticism relating to clinical conditions. Self‐criticism seemed to be a robust vulnerability factor for depression. Whereas self‐criticism displayed negative associations with antisocial behaviour, general health and social functioning, dependency showed certain protective effects. In terms of anxiety, obsessive‐compulsive disorder and present suicidal ideation, we observed the gender incongruence effect of dependency and self‐criticism. Such findings not only indicate the value of considering adolescent personality vulnerabilities in both psychopathology research and clinical practice, but also suggest the need for further studies to investigate the mechanisms underlying the observed difference in terms of the associations of dependency and self‐criticism with different mental disorders.

## AUTHOR CONTRIBUTIONS

**Yushi Bai:** Conceptualization; methodology; formal analysis; visualization; writing – original draft; writing – review and editing; data curation; software; validation. **Nick Midgley:** Writing – review and editing; supervision; validation. **Patrick Luyten:** Conceptualization; writing – review and editing; supervision; validation; methodology.

## CONFLICT OF INTEREST STATEMENT

The authors declare no conflicts of interest.

## Supporting information


Data S1:

## Data Availability

The data are not publicly available due to privacy and ethical restrictions. Sharing of data will require a data sharing agreement. The research group can provide descriptive data in table form or data analysis code.
