# Supplementary material for: Self‐criticism and dependency in adolescents with depression: Associations with clinical features and psychological functioning
Source: Br J Clin Psychol. 2026 Feb 23;65(2):639–55. doi: 10.1111/bjc.70038 (PMC13159778; doi:10.1111/bjc.70038)
Supplement: Supplementary file 1 — Data S1: [file BJC-65-639-s001.docx]

**Supplementary Materials for: Self-criticism and Dependency in Adolescents with Depression: Associations with Clinical Features and Psychological Functioning**

**Supplementary Appendix S1: Confirmatory Factor Analysis for the Short Version of the Depressive Experiences Questionnaire – Adolescent version (DEQ-A)**

***Statistical Analytical Procedure***

Confirmatory factor analysis (CFA) was used to test the factor structure of the short version of the Depressive Experiences Questionnaire – Adolescent version (DEQ-A). Maximum likelihood estimation was used in the CFA. Two models were tested and compared: (1) a theory-based parallel two-factor model with correlated structures of dependency and self-criticism; and (2) a one-factor model with all items loading on a general factor. A hierarchical model with lower-order factors (e.g., self-criticism and dependency) loading on to higher-order factors was excluded from consideration for two reasons: first, the model with two correlated first-order factors is mathematically equal to the model with two first-order factors loading on one second-order factor (Hau et al., 2004); secondly, there are potential identification/estimation flaws in the model with only two first-order factors loading on one second-order factor (Kline, 2015).

The evaluation and comparison of model fits were based on the multiple criteria of the 7 goodness-of-fit index following the suggestions of Hu and Bentler (1999) and Bentler and Bonett (1980). For the absolute fit index, the current study used the root mean square error of approximation (RMSEA), and a value smaller than .60 was regarded as a reasonable fit (Hu & Bentler, 1999). The classic index of Chi-square goodness-of-fit was excluded as this statistic is sensitive to sample size; that is, that it tends to reject reasonable models with large sample sizes but accepts poor models if the sample size is rather small (Kline, 2015). Therefore, the alternative index of the ratio of the Chi-square to the degrees of freedom (*χ^2^/df*) was used, and a ratio lower than 3 was regarded as an acceptable level (Schreiber et al., 2006). The Bender’s comparative fit index (CFI) and Tucker–Lewis index (TLI) were used as incremental fit indices to indicate the model fit. A value higher than .90 was regarded as an indicator of a reasonable model for both CFI and TLI (Bentler & Bonett, 1980; Van de Schoot et al., 2012). The Akaike information criterion (AIC) and the expected cross-validation index (ECVI) were referred to in order to compare the quality of non-nested models. Smaller values of ACI and ECVI indicate better model quality (Schreiber et al., 2006). Refinement of the models was carefully conducted based on the modification indices and the theoretical framework.

Once a basic model with an adequate model fit was obtained, multigroup CFA was conducted to test the measurement invariance between the sexes. Three levels of measurement invariance were tested: configural invariance, metric invariance, and scalar invariance (Putnick & Bornstein, 2016). Configural invariance implies that the same model structure is valid across groups (e.g., sex groups). This level of invariance was regarded as being achieved if the same model structure demonstrated a reasonable model fit across the sexes. Metric invariance consists of the equivalent factor loadings across groups. The metric invariance was tested by comparing the unconstrained model with the model constrained factor loadings. The Chi-square difference test was adopted to compare nested models, with a non-significant result indicating no meaningful difference between models and supporting metric invariance. Scalar invariance indicates that factor loadings and residuals are equivalent across groups. Again, the Chi-square test was used to compare the unconstrained model with the model-constrained factor loadings and intercepts, with a non-significant result indicating passing scalar invariance. Following the recommendations of Steenkamp and Baumgartner (1998) and Vandenberg and Lance (2000), partial invariance was accepted if more than half of the items under one factor were invariant. Obtaining the three levels of measurement invariance implies that factor scores from different groups demonstrate the same measurement unit and origin, thereby qualifying comparisons of factor means. Each factor of the final model was tested for internal consistency among the whole sample, and for boys and girls separately. While higher values of Cronbach’s α reflect better internal consistency, a value larger than .65 is often considered to be sufficient for a scale that is used to conduct research on human dimensions (Vaske, 2019; Vaske et al., 2017).

***Results and Brief Discussion***

The analysis was based on non-reversed data; reverse-worded items (items 4, 19, and 20) displayed negative loadings on their corresponding factors. The initial two-factor model demonstrated a rather poor model fit (Table S1). Yet, all paths had significant standardized factor loadings except the path from item 19 to self-criticism (Table S1). The path from item 19 to self-criticism, including the path from item 2 to dependency, were sequentially deleted from the initial two-factor model, as their standardized factor loadings were considerably low, with λ = –.050 and .163, respectively. To prevent purely statistically driven post-hoc model fitting, the model was refined based on both theoretical and statistical justifications. The modified model, with eight pairs of error items correlated, displayed fairly good model fit (*χ^2^/df* = 2.097, RMSEA = .053, CFI = .928, TLI = .904). It also had lower values in AIC (244.578 < 472.883) and ECVI (0.635 < 1.228) compared with the initial two-factor model. All the paths in the modified model had significant standardized factor loadings with the absolute values of λ ranging from .218 to .664 (Table S1). Dependency and self-criticism were highly correlated (r= .829). Therefore, a similar procedure was conducted for the estimation of a one-factor model. The initial one-factor model showed a poor model fit, with all of the paths demonstrating significant factor loadings (Table S1). Again, the paths from items 19 and 2 were deleted due to low factor loadings (λ = .008 and .142). The model was further refined by covarying 10 pairs of error items based on theoretical and statistical justifications. The modified model had a reasonable model fit, with *χ^2^/df* = 2.197 RMSEA = .056, CFI = .923, TLI = .895. Although the modified one-factor model showed lower values in AIC (251.205 < 524.209) and ECVI (0.652 < 1.362) compared with the initial one-factor model, it had higher values in AIC (251.205 > 244.578) and ECVI (0.652 > 0.635) compared with the modified two-factor model, indicating that the modified two-factor model had slightly better model fit. Therefore, the modified two-factor model was regarded as a final model, and its measurement invariance was further tested.

Table S1. Confirmatory Factor Analysis for Evaluating and Comparing Models of the short version of the Depressive Experiences Questionnaire – Adolescent version

|  |  | Two-Factor Model | |  | One-Factor model | |
| --- | --- | --- | --- | --- | --- | --- |
|  |  | Initial | Modified |  | Initial | Modified |
| Standardized regression weight | | |  |  |  |  |
|  | Q2 <D (or O) | .16** | – |  | .14* | – |
|  | Q5 <--D (or O) | .55*** | .57*** |  | .52*** | .49*** |
|  | Q9 <--D (or O) | .56*** | .63*** |  | .58*** | .59*** |
|  | Q11 <--D (or O) | .45*** | .38*** |  | .41*** | .38*** |
|  | Q13 <--D (or O) | .43*** | .35*** |  | .41*** | .38*** |
|  | Q14 <--D (or O) | .58*** | .46*** |  | .51*** | .45*** |
|  | Q16 <--D (or O) | .47*** | .45*** |  | .45*** | .43*** |
|  | Q17 <--D (or O) | .63*** | .57*** |  | .58*** | .54*** |
|  | Q3 <--S (or O) | .42*** | .43*** |  | .44*** | .44*** |
|  | Q4 <--S (or O) | –.31*** | –.22*** |  | –.21*** | –.20*** |
|  | Q6 <--S (or O) | .67*** | .65*** |  | .58*** | .55*** |
|  | Q8 <--S (or O) | .50*** | .50*** |  | .45*** | .45*** |
|  | Q10 <--S (or O) | .66*** | .66*** |  | .64*** | .64*** |
|  | Q12 <--S (or O) | .36*** | .36*** |  | .35*** | .39*** |
|  | Q19 <--S (or O) | –.05 | – |  | .01 | – |
|  | Q20 <--S (or O) | –.51*** | –.46*** |  | –.40*** | –.41*** |
| Correlation | |  |  |  |  |  |
|  | D-S | .73*** | .83*** |  | – | – |
| Model fit | |  |  |  |  |  |
|  | *χ^2^/df* | 3.64 | 2.10 |  | 4.12 | 2.20 |
|  | RMSEA | .08 | .05 |  | .09 | .06 |
|  | CFI | .75 | .93 |  | .71 | .92 |
|  | TLI | .71 | .90 |  | .66 | .90 |
|  | AIC | 472.88 | 244.58 |  | 524.21 | 251.21 |
|  | ECVI | 1.23 | 0.64 |  | 1.36 | 0.65 |

*Note:* 1. * *p* < .05, ** *p* <.01, *** *p* < .001.

2. S = Self-criticism; D = dependency; O = one-factor; RMSEA = root mean square error of approximation; CFI = Bender’s comparative fit index; TLI = Tucker–Lewis index; AIC = Akaike information criterion; ECVI = expected cross-validation index.

Multigroup CFA for the modified two-factor model was conducted by dividing the sample by sex. The results for the multigroup CFA revealed an overall reasonable model fit across sex groups (*χ^2^/df* = 1.690, RMSEA = .042, CFI = .909, TLI = .878), implying passing configural invariance. The non-significant Chi-square difference test pointed to an equivalent model fit between unconstrained and metric models (*χ^2^* = 10.74, *p* = .706), which further suggested that metric invariance was achieved. However, the Chi-square difference test between unconstrained and scalar models was significant (*χ^2^* = 28.48, *p* = .010), highlighting an important change in model fit between the two models. The source of the non-invariance was then investigated by sequentially releasing item intercept constraints in a backward approach. After releasing the constraints for items 17 and 16, partial scalar invariance was achieved (*χ^2^* = 37.82, *p* = .063). The internal consistency of the modified two-factor model was then tested and found to be overall acceptable, as the Cronbach’s α for dependency was .721, .719, and .682 for the whole sample, for girls, and for boys, respectively, and the Cronbach’s α for self-criticism was .669, .652, and .696 for the whole sample, for girls, and for boys, respectively.

In summary, the evidence supports the validity of the two-factor structure of the short version of the DEQ-A. Moreover, the results from the measurement invariance test demonstrated that the concepts of dependency and self-criticism were consistent across the sexes, with the same measurement unit (i.e., factor loading) and origin (i.e., intercept). This substantiates the proposed need for further comparisons and analyses of dependency and self-criticism between sex groups. Each factor presented adequate internal consistency for the whole sample of girls and boys, supporting the reliability of applying the modified two-factor scale to the current sample. It should be noted that the correlation between the two latent variables of self-criticism and dependency was relatively high (*r* = .829) in the modified two-factor model. This is consistent with Blatt’s theoretical framework, as distortions in self-criticism and dependency have been assumed to develop in a synergistic and interactive manner (Blatt & Luyten, 2009). However, we also note that an overly high correlation between latent variables can be problematic, as it may indicate inadequate discriminant validity. Although the definition of ‘high correlation’ varies across sources, one commonly used criterion is the one proposed by Brown (2015), which notes that a correlation between latent variables of over .85 indicates poor discriminant validity. Indeed, although the correlation between the two constructs was .829 in the current study, the modified two-factor model still demonstrated better model fit compared with the unidimensional one-factor model. More importantly, by using the modified two-factor DEQ-A, the correlation and regression analysis detected distinct patterns in terms of dependency and self-criticism relating to clinical conditions and psychological functioning, which supports the discriminant validity of factors in the modified DEQ-A.

**Supplementary Appendix Table S2: Regressions Analysis for Variables Predicting Friendships**

|  |  | FQ | | |
| --- | --- | --- | --- | --- |
|  |  | *B* | *SE B* | *β* |
| SC |  | –0.71 | 1.32 | -.11 |
| D |  | –0.11 | 1.10 | -.02 |
| Sex |  | 0.64 | 1.28 | .05 |
| D×SC |  | –0.04 | 0.76 | -.01 |
| D×gender |  | 0.96 | 1.23 | .16 |
| SC×gender |  | –0.33 | 1.51 | -.04 |
| D×SC×gender |  | –0.70 | .94 | -.10 |
| *R^2^* |  | .03 | | |
| *F* |  | 0.75 | | |

*Note:* **p* < .05, ***p* < .001; B /*β*= unstandardized/standardized regression coefficient; *SE* = standard error; S/D = self-criticism/dependency from modified DEQ-A; FQ = Friendship Questionnaire.

**Supplementary Appendix Table S3: Regression Analysis for Variables Predicting Self-Criticism and Dependency**

|  | SC | | |  | D | | |
| --- | --- | --- | --- | --- | --- | --- | --- |
|  | *B* | *SE B* | *β* |  | *B* | *SE B* | *β* |
| FAD | 0.04 | 0.01 | **.29**** |  | 0.02 | 0.02 | .14 |
| APQ-PP | –0.00 | 0.03 | –.02 |  | 0.02 | 0.03 | .05 |
| APQ-ID | 0.01 | 0.03 | .02 |  | 0.03 | 0.03 | .10 |
| APQ-PS | 0.02 | 0.03 | .06 |  | 0.04 | 0.03 | .09 |
| *R^2^* | .11 | | |  | .05 | | |
| *F* | **5.40**** | | |  | 2.11 | | |

*Note:* **p* < .05, ***p* < .001; B /β= unstandardized/standardized regression coefficient; *SE* = standard error; S/D = self-criticism/dependency from modified DEQ-A; FAD = Family Assessment Device; APQ-PP/ID/PS = Alabama Parenting Questionnaire-Positive Parenting/Inconsistent Discipline/Poor Supervision.

**References**

Bentler, P. M., & Bonett, D. G. (1980). Significance tests and goodness of fit in the analysis of covariance structures. *Psychological Bulletin, 88*(3), 588–606 . https://psycnet.apa.org/doi/10.1037/0033-2909.88.3.588

Blatt, S. J., & Luyten, P. (2009). A structural–developmental psychodynamic approach to psychopathology: Two polarities of experience across the life span. *Development and Psychopathology*, *21*(3), 793–814. https://doi.org/10.1017/S0954579409000431

Brown, T. A. (2015). *Confirmatory factor analysis for applied research*. New York, NY: Guilford Publications.

Hau, K.-T., Wen, Z., & Cheng, Z. (2004). *Structural equation model and its applications*. Beijing, China: Educational Science.

Hu, L.-T., & Bentler, P. M. (1999). Cutoff criteria for fit indexes in covariance structure analysis: Conventional criteria versus new alternatives. *Structural Equation Modeling: A Multidisciplinary Journal, 6*(1), 1–55. https://doi.org/10.1080/10705519909540118

Jackson, D. L., Gillaspy, J. A., Jr, & Purc-Stephenson, R. (2009). Reporting practices in confirmatory factor analysis: An overview and some recommendations. *Psychological Methods, 14*(1), 6–23. https://doi.org/10.1037/a0014694

Kline, R. B. (2015). *Principles and practice of structural equation modeling*. New York, NY: Guilford Publications.

Putnick, D. L., & Bornstein, M. H. (2016). Measurement invariance conventions and reporting: The state of the art and future directions for psychological research. *Developmental Review, 41*, 7190. https://doi.org/10.1016/j.dr.2016.06.004

Schreiber, J. B., Nora, A., Stage, F. K., Barlow, E. A., & King, J. (2006). Reporting structural equation modeling and confirmatory factor analysis results: A review. *The Journal of Educational Research, 99*(6), 323–338. https://doi.org/10.3200/JOER.99.6.323-338

Steenkamp, J.-B. E. M., & Baumgartner, H. (1998). Assessing measurement invariance in cross-national consumer research. *Journal of Consumer Research, 25*(1), 78–90. https://doi.org/10.1086/209528

Van de Schoot, R., Lugtig, P., & Hox, J. (2012). A checklist for testing measurement invariance. *European Journal of Developmental Psychology, 9*(4), 486–492. https://doi.org/10.1080/17405629.2012.686740

Vandenberg, R. J., & Lance, C. E. (2000). A review and synthesis of the measurement invariance literature: Suggestions, practices, and recommendations for organizational research. *Organizational Research Methods, 3*(1), 4–70. https://psycnet.apa.org/doi/10.1177/109442810031002

Vaske, J. J. (2019). *Survey research and analysis*. Urbana, IL: Sagamore-Venture.

Vaske, J. J., Beaman, J., & Sponarski, C. C. (2017). Rethinking internal consistency in Cronbach’s alpha. *Leisure Sciences, 39*(2), 163–173. https://psycnet.apa.org/doi/10.1080/01490400.2015.1127189
